# Kinase Gene Expression Profiling of Metastatic Clear Cell Renal Cell Carcinoma Tissue Identifies Potential New Therapeutic Targets

**DOI:** 10.1371/journal.pone.0160924

**Published:** 2016-08-30

**Authors:** Pooja Ghatalia, Eddy S. Yang, Brittany N. Lasseigne, Ryne C. Ramaker, Sara J. Cooper, Dongquan Chen, Sunil Sudarshan, Shi Wei, Arjun S. Guru, Amy Zhao, Tiffiny Cooper, Deborah L. Della Manna, Gurudatta Naik, Richard M. Myers, Guru Sonpavde

**Affiliations:** 1 Department of Internal Medicine, University of Alabama at Birmingham (UAB), Birmingham, AL, United States of America; 2 Department of Radiation Oncology, UAB, Birmingham, AL, United States of America; 3 HudsonAlpha Institute for Biotechnology, Huntsville, AL, United States of America; 4 Department of Genetics, UAB, Birmingham, AL, United States of America; 5 UAB Department of Preventive Medicine, Birmingham, AL, United States of America; 6 UAB Department of Urology, Birmingham, AL, United States of America; 7 UAB Department of Urologic Pathology, Birmingham, AL, United States of America; 8 UAB Department of Medicine, Section of Hematology-Oncology and the UAB Comprehensive Cancer Center, Birmingham, AL, United States of America; University of Tennessee Health Science Center, UNITED STATES

## Abstract

Kinases are therapeutically actionable targets. Kinase inhibitors targeting vascular endothelial growth factor receptors (VEGFR) and mammalian target of rapamycin (mTOR) improve outcomes in metastatic clear cell renal cell carcinoma (ccRCC), but are not curative. Metastatic tumor tissue has not been comprehensively studied for kinase gene expression. Paired intra-patient kinase gene expression analysis in primary tumor (T), matched normal kidney (N) and metastatic tumor tissue (M) may assist in identifying drivers of metastasis and prioritizing therapeutic targets. We compared the expression of 519 kinase genes using NanoString in T, N and M in 35 patients to discover genes over-expressed in M compared to T and N tissue. RNA-seq data derived from ccRCC tumors in The Cancer Genome Atlas (TCGA) were used to demonstrate differential expression of genes in primary tumor tissue from patients that had metastasis at baseline (n = 79) compared to those that did not develop metastasis for at least 2 years (n = 187). Functional analysis was conducted to identify key signaling pathways by using Ingenuity Pathway Analysis. Of 10 kinase genes overexpressed in metastases compared to primary tumor in the discovery cohort, 9 genes were also differentially expressed in TCGA primary tumors with metastasis at baseline compared to primary tumors without metastasis for at least 2 years: *EPHB2*, *AURKA*, *GSG2*, *IKBKE*, *MELK*, *CSK*, *CHEK2*, *CDC7* and *MAP3K8*; p<0.001). The top pathways overexpressed in M tissue were pyridoxal 5'-phosphate salvage, salvage pathways of pyrimidine ribonucleotides, NF-kB signaling, NGF signaling and cell cycle control of chromosomal replication. The 9 kinase genes validated to be over-expressed in metastatic ccRCC may represent currently unrecognized but potentially actionable therapeutic targets that warrant functional validation.

## Background

Clear cell renal cell carcinoma (ccRCC) accounts for approximately 70–80% of kidney cancers [1]. At the time of diagnosis, 30% of RCC patients have metastatic lesions, and 30–50% will develop metastases during follow up after definitive surgery for localized disease. Metastatic ccRCC is generally incurable and long-term survival is observed in less than 10% of patients. While high-dose interleukin (IL)-2 remains an option due to durable complete remissions in ~7% of patients, toxicities preclude its administration in the vast majority of patients [2, 3]. Protein kinases are central-signaling molecules that affect proliferation, differentiation, motility, cell death and survival [4]. Several kinase inhibitors, including vascular endothelial growth factor receptor (VEGF) receptor tyrosine kinase inhibitors (TKIs), sunitinib, sorafenib, axitinib, pazopanib and cabozantinib, and mammalian target of rapamycin (mTOR) inhibitors, temsirolimus and everolimus, improve outcomes in metastatic RCC [5–11]. More recently, nivolumab, a programmed death (PD)-1 inhibitor immunotherapeutic, cabozantinib, a multitargeted kinase inhibitor, and the combination of lenvatinib plus everolimus, improved outcomes in patients progressing after treatment with VEGF inhibitors [11–13]. However, these agents are not curative, and intrinsic or acquired resistance result in a median overall survival of 2 to 3 years. Hence, a better understanding of tumor biology and new drug targets are critical to further advance ccRCC treatments.

Molecular analyses of tumor tissue have been used to address these needs. Comprehensive molecular profiling of many cancers, including ccRCC, has employed primary tumor tissue, as typified by The Cancer Genome Atlas (TCGA) Project and other smaller studies [14–16]. The tumor suppressor genes, SETD2, BAP1, and PBRM1 have been shown to be altered in addition to the VHL gene, although their therapeutic significance is unknown [17]. Although the PI3K pathway was recurrently mutated, the utility of inhibitors of this pathway heretofore has been modest [18, 19]. Few genomics studies of small numbers of patients have analyzed exome sequencing of metastatic ccRCC tumor tissue, but these have not focused on actionable molecules or studied gene expression [20, 21]. Kinase targets are particularly relevant because their inhibitors are both already approved for use in multiple malignancies and can be readily designed, facilitating therapeutic targeting[22]. In this study, we examined differential expression of kinase genes in matched and paired primary tumor tissue (T), adjacent normal kidney (N) and metastatic tumor tissue (M) from 35 ccRCC patients. For this work, we took advantage of the low quantity of nucleic acid material required by the NanoString nCounter platform to quantify gene expression from widely available formalin-fixed paraffin embedded (FFPE) tissue *[23–27].* Furthermore, we explored evidence for the importance of these genes in the independent dataset from TCGA [14].

## Materials and Methods

### Patient and tumor selection

Treatment-naïve patients who underwent nephrectomy (total or partial) at UAB for ccRCC between years 2000 to 2013 were identified for the discovery dataset. Medical records were abstracted and clinical data including age at diagnosis, stage, tumor grade and metastatic tissue site were recorded. De-identified patients with available FFPE tissue from the metastatic tumor (M), primary tumor (T) and adjacent normal (N) kidney tissue were selected. Patients with bone as the metastatic tumor tissue site were excluded as demineralization during processing affects measurement of gene expression. The study was approved by the UAB Institutional Review Board (IRB).

### Tumor dissection and RNA extraction

The tissue underwent central pathology review by a urologic pathologist. Tumor with predominantly clear cell component was demarcated for histologic macrodissection performed on 10 μm sections. RNA was isolated from dissected primary and metastatic tumor and normal kidney tissue using RNeasy FFPE kit (Qiagen, Valencia, CA). A tissue surface area of approximately 100 mm^2^ is adequate to harvest the necessary amount of RNA (~100 ng). RNA integrity was assessed via the 260/280 ratio using nanodrop.

### NanoString platform for gene expression assay

RNA is input directly into the nCounter^TM^ platform (NanoString Technologies, Seattle, WA) for the hybridization reaction containing color-coded molecular barcodes representing the gene 519 kinase genes and 8 internal reference genes (S1 Table) [23, 28]. A codeset specific to a 100-base region of the target mRNA was custom designed by NanoString Technologies using a 3’ biotinylated capture probe and a 5’ reporter probe tagged with a specific fluorescent barcode, creating two sequence-specific probes for each target transcript. Probes were hybridized to 100ng of total RNA for 19 hours at 65°C and then applied to the nCounter^TM^ preparation station for automated removal of excess probe by immobilization of probe-transcript complexes on a streptavidin-coated cartridge.

### Data processing

Data were collected using the nCounter^TM^ Digital Analyzer by counting the individual barcodes.

Each codeset included probes for the 519 kinase genes, spiked-in external RNA consortium positive and negative controls, and 8 reference housekeeping genes. Background hybridization was determined using spiked-in negative controls. All signals below mean background plus 2 standard deviations were considered to be below the limits of detection, and set to mean background. A normalization factor was calculated from the spiked-in exogenous positive controls in each sample and applied to the raw counts from the nCounter^TM^ output data.

### Statistical and pathway analysis

The mean signals from N, T and M tissue were used to calculate change in gene expression, and a p-value by t-test <0.05 was considered significant. Change in mean intensity between tumor and metastatic site was used to derive top 10 kinases overexpressed in metastases compared to primary tumors. Functional analysis was conducted to identify key signaling pathways by using Ingenuity Pathway Analysis. The genes that had statistically significant (p<0.05) overexpression in the metastatic site vs primary tumor site were used for pathways analysis. Genes that were also significantly overexpressed in T vs. N tissue were eliminated in order to isolate kinases over-expressed in metastases only.

### Heatmap generation

**Fig 1:**
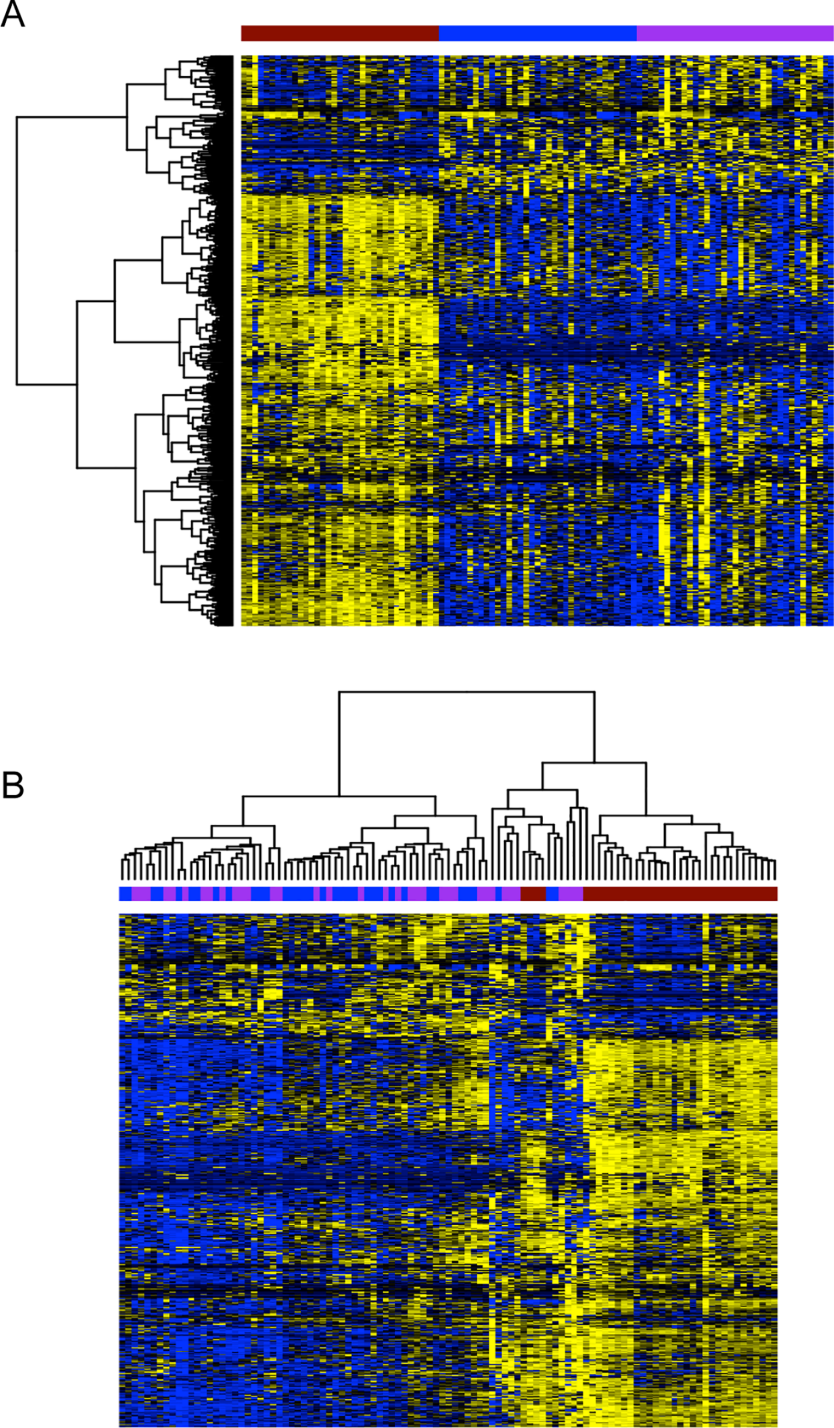
Clustering of kinase genes showing A) normal tissue on the left (N, red), tumor in the middle (T, blue) and metastatic tumor tissue on the right (M, purple), and B) hierarchical clustering of all normal (N, red), tumor (T, blue), and metastatic (M, purple) tissues. Figure legend: X-axis represents the tissue sample, Y-axis represents the kinases. Yellow: up regulated kinases, Blue: down regulated kinases.

Fig 1 was generated in R (version 3.2.1) with RStudio (version 0.99.467, http://www.rstudio.com/). All genes with a normalized standard deviation less than 2 were removed and data was scaled using the scale() function before plotting. Heatmaps were generated with the heatmap.2 function with method = “ward.D2” from the gplots (version 2.17.0) package.

### External validation in the TCGA dataset

For external validation, we downloaded the TCGA RNA-seq (UNC IlluminaHiSeq_RNASeqV2) dataset Level 3 results for all ccRCC patients on April 30, 2015 from http://cancergenome.nih.gov/. Primary tumors were defined as non-metastatic (M0) or metastatic (M1) based on stage at the time of entry to TCGA. Patients who had metastasis at baseline were compared with those that did not develop metastasis for at least 2 years for our primary external validation analysis. Secondarily, we also performed the following 2 comparisons: 1) those who developed metastasis vs. those who did not develop metastasis for at least 2 years, and 2) those who had no metastasis at baseline vs. those that did.

Analysis was conducted in R (version 3.2.1) with RStudio (version 0.99.467, http://www.rstudio.com/). Gene expression changes were examined by the R package DESeq2 (version 1.6.3), a method for differential analysis of sequence read count data [29]. All analysis used default settings, but employed likelihood ratio test (LRT) hypothesis testing, and removed non-convergent transcripts from subsequent analysis. False discovery rates (FDR) were estimated using the Benjamini-Hochberg (BH) algorithm[30].

## Results

### Patient population

For our discovery study, we identified 35 patients with adequate metastatic ccRCC tumor tissue (M), primary tumor (T) and adjacent normal (N) kidney. Adequate tissue was defined as a minimum of 100 mm^2^ of tumor in sections of 10 μM thickness. Clinical characteristics, including age at diagnosis, patient sex, patient race, nephrectomy type, clinical stage at diagnosis, Fuhrman grade, histology, and metastatic organ sites for this cohort are detailed in Table 1. The most common metastatic tumor sites were lungs and soft tissue/nodal followed by other visceral sites.

**Table 1:** Patient and tumor characteristics.

Characteristic	No. of patients (%)
• Age at diagnosis, yearsMedianRange	• 5636–77
Sex	• 30 (86%)5 (14%)
MaleFemale	
• RaceCaucasianAfrican AmericanHispanicOther	• 28 (80%)1 (2.8%)1 (2.8%)5 (14.2%)
• Type of nephrectomyComplete Partial	• 31 (88.5%)4 (11.4%)
• Clinical stage at diagnosis1234	• 3 (8.6%)6 (17%)11 (31.4%)15 (42.8%)
• Fuhrman grade1234	• 1 (2.8%)14 (40%)15 (42.8%)5 (14.3%)
• HistologyClear cell onlyClear cell with rhabdoidClear cell with sarcomatoidClear cell with papillaryClear cell with granular	• 29 (82.8%)1 (2.8%)3 (8.6%)1 (2.8%)1 (2.8%)
• Metastatic organ sites for studyLungs Soft tissue or lymph nodeAdrenal glandBrainPancreasLiver	• 13 (37.1%)10 (28.6%)5 (14.2%)4 (11.4%)2 (5.6%)1 (2.8%)

### Kinase gene expression in metastatic tumor and pathway analysis

Unsupervised and supervised clustering of the 519 kinase genes (S2 Table) showed increased gene expression of several kinase genes in M tumors compared to T tumors and N adjacent renal tissue (Fig 1A and 1B). The top 10 kinase genes over-expressed in M compared to T and N tissue (Table 2) were *EPHB2*, *AURKA*, *GUCY2C*, *GSG2*, *IKBKE*, *MELK*, *CSK*, *CHEK2*, *CDC7* and *MAP3K8* (p<0.05). A total of 33 genes exhibited >1.7 fold increased expression in M vs. T or N in at least a third of the 35 (n≥12) patients (Table 3). Using Ingenuity Pathway Analysis, the top pathways identified based on the kinases overexpressed in M compared to N or T tissue were pyridoxal 5'-phosphate salvage, salvage pathways of pyrimidine ribonucleotides, NF-kB signaling, NGF signaling, and cell cycle control of chromosomal replication (Table 4).

**Table 2:** Top kinase genes overexpressed in all metastases compared to all primary ccRCC tumors in 35 patients from the discovery dataset.

Gene name	Metastatic tumor mean	Primary tumor mean	Fold change	P VALUE (Metastatic vs primary tumor)
EPHB2	15.06	5.63	2.67	0.011
AURKA	24.79	14.11	1.75	0.000
GUCY2C*	2.22	1.27	1.75	0.023
GSG2	3.75	2.34	1.60	0.006
IKBKE	66.02	41.29	1.59	0.025
MELK	57.23	35.94	1.59	0.005
CSK	382.90	241.12	1.59	0.031
CHEK2	47.63	30.43	1.56	0.001
CDC7	19.58	12.51	1.56	0.031
MAP3K8	144.50	95.63	1.51	0.011

**Table 3:** Top kinase genes overexpressed >1.7 fold in metastases in > a third of patients in all metastases compared to all primary ccRCC tumors in 35 patients from the discovery dataset.

No. of patients (%)	Genes
18 (51.4%)	ADCK1, DYRK4, PDGFRA, PRKCA, ROR2, TYRO3
17 (48.5%)	TEC, PLK4, EPHB3
16 (45.7%)	PLK1, NEK2, EPHB2, EPHA3, CKD1
15 (42.8%)	AURKA, ROS1, PHMYT1, PASK, MAPK11, Kit, BUB1B
14 (40%)	IRAK3, CHEK1, AATK
13 (37.1%)	PRKCE, CAMK4, BUB1
12 (34.2%)	PRKCQ, MAPK12, KALRN, IKBKE, FES, CDC7, BTK, BLK

**Table 4:** Top signaling pathways overexpressed in all metastases compared to all primary ccRCC tumors in 35 patients from the discovery dataset.

Ingenuity Canonical Pathways	Kinases
Pyridoxical 5’-Phosphate Salvage Pathway	EIF2AK2, IRAK1, MAP3K8, LIMK1
Salvage Pathways of Pyrimidine Ribonucleotides	EIF2AK2, IRAK1, MAP3K8, LIMK1
NK-kB signaling	EIF2AK2, IRAK1, MAP3K8, INSR
NGF signaling	MAP3K8, TRIO, IKBKE
Cell cycle control of chromosomal replication	CDC7, CHEK2

### Kinase gene overexpression in primary tumors from patients in the TCGA dataset with metastatic vs. non-metastatic ccRCC

To examine our findings in an independent cohort, we analyzed RNA-seq data from the TCGA ccRCC cohort, which includes kinase gene expression data in both primary tumor (n = 497) and adjacent benign (n = 72) kidney tissue. First we compared primary tumor kinase gene expression in patients with metastasis at baseline (n = 79) to patients without metastasis for at least 2 years (n = 187). Nine of the 10 significant kinase genes identified in our discovery study were significantly overexpressed in the primary tumors of patients with metastasis at baseline compared to primary tumors in patients without metastases for at least two years (adjusted p<0.001). Although *GUCY2C* was not significantly differentially expressed between these groups, a closer examination reveals low levels of expression in both the discovery and validation cohorts, which may explain our inability to validate it in the second cohort (Table 5). When comparing patients with localized disease at baseline who subsequently developed metastasis (n = 28) to patients who did not develop metastasis for at least 2 years (n = 187), 8 of the 10 kinase genes were significantly over-expressed in primary tumors from metastatic patients (adjusted p <0.05), with *CSK and MAP3k8* being the exceptions. When comparing primary tumors in patients with no metastasis at baseline (n = 418) to primary tumors with metastasis at baseline (n = 79), again, with the exception of *GUCY2C*, the other 9 kinase genes were significantly overexpressed (adjusted p<0.05). These 9 kinases were examined for alterations in DNA sequence or copy number and no significant alterations were identified (data not shown). Alterations at the protein level of these kinases could not be examined since they were not analyzed by TCGA.

**Table 5:** Expression of kinase genes in primary tumors from metastatic vs. non-metastatic patients in the validation dataset*.

Genes	baseMean	p-value	adjusted p-value
MELK.9833	199.5619451	8.74E-18	2.80E-15
GSG2.83903	46.98529174	1.87E-15	3.30E-13
CHEK2.11200	307.6903398	3.99E-11	2.87E-09
AURKA.6790	353.7879497	3.39E-10	1.85E-08
IKBKE.9641	496.7497848	3.98E-10	2.12E-08
EPHB2.2048	389.0931124	1.69E-09	7.51E-08
CDC7.8317	251.725834	2.29E-05	0.000202497
MAP3K8.1326	480.1769944	0.000472431	0.002394953
CSK.1445	3162.71356	0.001042731	0.004538433
GUCY2C.2984	7.891334454	0.691714419	0.772671281

*comparing expression of top 10 discovery dataset kinase genes in TCGA patients who had metastasis at baseline (n = 79) vs. those who did not develop metastasis for at least 2 years (n = 187); baseMean (DESeq2 generated normalized mean count); *GUCY2C was the only kinase gene that was not externally validated in the TCGA dataset, but GUCY2C.2984 had a very low mean count in the TCGA dataset (8.6), as well as in the discovery dataset (2.22).

We also evaluated the TCGA dataset for gene expression changes of the 33 kinase genes with >1.7 fold increased expression in M vs. T in at least a third of patients in our discovery dataset. We found when comparing primary tumors in patients with metastasis at baseline (n = 79) to primary tumors in patients who did not develop metastasis for at least 2 years (n = 187), 21 of 33 kinase genes were differentially expressed (adjusted p<0.05) (S3 Table). When comparing primary tumors who developed metastasis (n = 28) vs. those who did not develop metastasis for at least 2 years (n = 187) (S4 Table), or when comparing those who had no metastasis at baseline (n = 418) vs. those that did (n = 79), 18 and 19, respectively, of the 33 kinase genes were significantly over-expressed in metastatic patients (adjusted p<0.05) (S5 Table).

## Discussion

Molecular profiling of metastatic tumor tissue may be critical for making further advances in the therapy of metastatic ccRCC. Given that most studies have focused on profiling primary renal tumors and because kinases may be therapeutically actionable, we analyzed FFPE tissue from the intra-patient primary tumor, benign adjacent normal kidney and metastases to explore whether kinase genes are differentially expressed. Of the 10 kinase genes overexpressed in metastases compared to primary tumor in the discovery cohort, we demonstrated 9 genes were also differentially expressed in TCGA primary tumors with metastasis at baseline (n = 79) compared to primary tumors without metastasis for at least 2 years (n = 187). These 9 kinases are not currently readily recognized as therapeutic targets in ccRCC and include *EPHB2*, *AURKA*, *GSG2*, *IKBKE*, *MELK*, *CSK*, *CHEK2*, *CDC7* and *MAP3K8*. The study also highlights the substantial molecular heterogeneity of this disease, as no single kinase gene appeared dominant [14, 20, 21]. The targeted assay for gene expression made possible accurate quantification of kinases from small blocks of tissue with high sensitivity and accuracy.

Many of the validated 9 kinases have previously been implicated data in the metastatic progression of other malignancies. Moreover, inhibitors of some of these kinases are already in early phase I trials. *EphB2* is important in cell motility, epithelial mesenchymal transition (EMT), adhesion and angiogenesis, and appears prognostic in multiple other malignancies, especially colorectal cancers [31–37]. *AURKA* plays a role in chromosome segregation during mitosis and is correlated with mTOR pathway overexpression in sarcomatoid RCC [38]. GSG2 is required for mitotic histone phosphorylation and metaphase chromosome alignment [39]. Additionally, a preclinical study reported a potential role for GSG2 inhibitors in multiple malignancies [40]. IKBKE is a member of the nuclear factor-kappaB (NF-κB) signaling pathway and expression predicted poor/reduced survival in one study of ccRCC patients [41]. Another meta-analysis suggested that NF-κB may represent a therapeutic target in ccRCC [42]. MELK is thought to bind and activate transcription factors c-JUN and FOXM1 and play a role in maintaining tumor initiating cells [43–46]. Over-expression of CSK-binding protein appeared to contribute to renal cell carcinogenesis in a preclinical study [47]. CHEK2 is activated in response to DNA damage, and is involved in cell cycle arrest, but its role in ccRCC has not been studied [48]. Similarly, CDC7 (essential for the G1/S transition and initiation of DNA replication) and MAP3K8 (which activates MAPK and JNK) have not been validated as therapeutic targets in ccRCC and warrant further study. Notably, widely targeted kinases in other malignancies including EGFR/HER2 family and PI3K/mTOR pathway kinases were among those measured, but not found to be differentially expressed. However, when examining the 9 validated genes and other genes with >1.7-fold increase in M in at least a third of patients, agents are either approved for other indications (e.g. *BTK*, *MAPK and ROS1 inhibitors)* or undergoing investigation in phase I trials (e.g. *AURKA*, *CHEK1*, *CHEK2* and *PLK1 inhibitors)*, suggesting that clinical validation of their potential actionability may be feasible. Although our study focused on kinases, pathway analysis showed significance of salvage pathways of pyridoxal 5'-phosphate and pyrimidine ribonucleotides, which accords with the importance of a metabolic shift demonstrated by TCGA [14].

Although our study is limited by the size of the discovery dataset (n = 35), using paired intra-patient T, N and M samples is a strength. Additionally, because fresh frozen tumors are not readily available, we used FFPE tumor tissue. RNA extracted from FFPE material is typically highly degraded, but the nCounter^TM^ technology largely overcomes this obstacle [49]. Although genomic material may undergo degradation with age, all tumors in this study were obtained from biopsies performed after the year 2000. The TCGA validation cohort differs from our discovery cohort in two important ways. First, the discovery cohort employed NanoString, while the validation dataset used RNA-seq. However, these different but robust platforms assess gene expression through independent techniques and yielded highly concordant results in different patient cohorts. Second, while the TCGA validation cohort did not directly measure metastatic tissues, kinases were overexpressed in primary tumors with metastases compared to primary tumors without metastases. Future studies can further corroborate our results in a larger dataset of metastatic FFPE tumors. A limitation is that tumor heterogeneity leads to the presence of different clonal populations within the same primary or metastatic mass displaying differing kinase gene expressions, although our sample size of 35 patients may be expected to partly overcome this confounding factor and discover broadly important overexpressed genes in metastases. Another important future direction is to further investigate the activity and expression of the corresponding kinase proteins. While kinase gene expression may correlate with corresponding kinase protein levels and activity, many kinases are regulated at the translational or post-translational modification levels. Immunohistochemistry (IHC) could be performed to assay protein expression, but will likely require fresh frozen tissues because this platform is characterized by methodological and analytical limitations when performed on FFPE tissue. Inadequate tissue precluded performing quantitative polymerase chain reaction (PCR) to validate gene expression using a different platform and IHC to confirm expression of the corresponding proteins.

Nevertheless, our study has identified altered gene expression in kinase transcripts measured by two different platforms in two independent cohorts. These transcripts have broad deterministic ability to drive metastatic ccRCC as opposed to stochastic alterations important in smaller subsets of tumors. It is also possible that the 9 validated overexpressed kinase genes may be ‘shared’ among metastatic sites across patients, as opposed to being ‘privately’ overexpressed in small subsets of patients or metastatic sites. Furthermore, the expression of these genes may assist in improving the prognostic stratification of patients with localized ccRCC following surgery. Notably, other molecular panels such as the recurrence score and MET gene polymorphisms have been previously reported to improve prognostication of localized ccRCC [50, 51]. We could not evaluate the prognostic impact of kinase gene expression on outcomes with systemic therapy for metastatic disease because information on systemic therapy was unavailable. Moreover, this objective is beyond the scope of the study since the sample size is modest and such an analysis would be under-powered.

The only kinase gene expression change that was not externally validated in TCGA was *GUCY2C*, which had low absolute expression in both the discovery cohort and the TCGA cohort. Nevertheless, *GUCY2C* gene expression by microarray was associated with increasing metastatic potential in a study of primary ccRCC tumors, suggesting that further study of this gene in a larger dataset of metastatic tumor tissue is warranted [52]. Examining genes where more than a third of patients had >1.7 fold increase in expression between M vs. T sites, we observed a significant kinase gene expression increase in TCGA primary tumors with metastases at baseline compared to primary tumors in patients who did not develop metastasis for at least 2 years in 21 of the 33 genes identified in the discovery dataset. While differential gene expression changes in the TCGA cohort are not as significant in this set of kinases as the panel of 10 kinases overexpressed in all M compared to T tumors, this is not surprising given that this group of genes had a modest expression change in only a subset of patients.

## Conclusions

This study is the first to examine intra-patient kinase gene expression in ccRCC metastatic and primary tumor tissues and identifies nine over-expressed kinase genes (*EPHB2*, *AURKA*, *GSG2*, *IKBKE*, *MELK*, *CSK*, *CHEK2*, *CDC7* and *MAP3K8*; p<0.001) in metastatic ccRCC tumor tissue, which may represent drivers of disease and novel therapeutic targets. Our results remain hypothesis-generating and require further validation to confirm functional significance of findings. Preclinical activity of inhibitors of the identified kinases needs to be demonstrated to support clinical trials. Collaboration between institutions to comprehensively study a large dataset of metastatic ccRCC tissue will enable better understanding of this disease and inform both rational drug development and patient selection for targeted agents.

## Supporting Information

S1 Table NanoString panel of 519 kinase genes and 8 internal reference genes. (XLS)

S2 Table Nanostring raw data. (XLSX)

S3 Table Comparison of expression of all kinase genes between primary tumors from metastatic and non-metastatic patients. (DOCX)

S4 Table Comparison of expression of all kinase genes between primary tumors from patients without metastasis at resection and did not recur vs. those who recurred. (DOCX)

S5 Table Comparison of kinase gene expression between primary tumors from metastatic and non-metastatic patients at baseline. (DOCX)

## References

[1] Cohen HT, McGovern FJ. Renal-cell carcinoma. N Engl J Med. 2005;353(23):2477–90. Epub 2005/12/13. doi: 353/23/2477 [pii] 10.1056/NEJMra043172 . 16339096

[2] McDermott DF, Regan MM, Clark JI, Flaherty LE, Weiss GR, Logan TF, et al Randomized phase III trial of high-dose interleukin-2 versus subcutaneous interleukin-2 and interferon in patients with metastatic renal cell carcinoma. Journal of clinical oncology: official journal of the American Society of Clinical Oncology. 2005;23(1):133–41. Epub 2004/12/31. doi: 23/1/133 [pii] 10.1200/JCO.2005.03.206 . 15625368

[3] Fyfe G, Fisher RI, Rosenberg SA, Sznol M, Parkinson DR, Louie AC. Results of treatment of 255 patients with metastatic renal cell carcinoma who received high-dose recombinant interleukin-2 therapy. Journal of clinical oncology: official journal of the American Society of Clinical Oncology. 1995;13(3):688–96. Epub 1995/03/01. . 7884429 10.1200/JCO.1995.13.3.688

[4] Vlahovic G, Crawford J. Activation of tyrosine kinases in cancer. Oncologist. 2003;8(6):531–8. . 14657531 10.1634/theoncologist.8-6-531

[5] Motzer RJ, Hutson TE, Tomczak P, Michaelson MD, Bukowski RM, Rixe O, et al Sunitinib versus interferon alfa in metastatic renal-cell carcinoma. The New England journal of medicine. 2007;356(2):115–24. Epub 2007/01/12. doi: 356/2/115 [pii] 10.1056/NEJMoa065044 . 17215529

[6] Escudier B, Eisen T, Stadler WM, Szczylik C, Oudard S, Siebels M, et al Sorafenib in advanced clear-cell renal-cell carcinoma. The New England journal of medicine. 2007;356(2):125–34. 10.1056/NEJMoa060655 . 17215530

[7] Rini BI, Escudier B, Tomczak P, Kaprin A, Szczylik C, Hutson TE, et al Comparative effectiveness of axitinib versus sorafenib in advanced renal cell carcinoma (AXIS): a randomised phase 3 trial. Lancet. 378(9807):1931–9. Epub 2011/11/08. S0140-6736(11)61613-9 [pii] 10.1016/S0140-6736(11)61613-9 . 22056247

[8] Sternberg CN, Davis ID, Mardiak J, Szczylik C, Lee E, Wagstaff J, et al Pazopanib in locally advanced or metastatic renal cell carcinoma: results of a randomized phase III trial. Journal of clinical oncology: official journal of the American Society of Clinical Oncology. 2010;28(6):1061–8. 10.1200/JCO.2009.23.9764 . 20100962

[9] Hudes G, Carducci M, Tomczak P, Dutcher J, Figlin R, Kapoor A, et al Temsirolimus, interferon alfa, or both for advanced renal-cell carcinoma. The New England journal of medicine. 2007;356(22):2271–81. Epub 2007/06/01. 356/22/2271 [pii] 10.1056/NEJMoa066838 . 17538086

[10] Motzer RJ, Escudier B, Oudard S, Hutson TE, Porta C, Bracarda S, et al Efficacy of everolimus in advanced renal cell carcinoma: a double-blind, randomised, placebo-controlled phase III trial. Lancet. 2008;372(9637):449–56. 10.1016/S0140-6736(08)61039-9 . 18653228

[11] Choueiri TK, Escudier B, Powles T, Mainwaring PN, Rini BI, Donskov F, et al Cabozantinib versus Everolimus in Advanced Renal-Cell Carcinoma. The New England journal of medicine. 2015;373(19):1814–23. 10.1056/NEJMoa1510016 . 26406150 PMC5024539

[12] Motzer RJ, Escudier B, McDermott DF, George S, Hammers HJ, Srinivas S, et al Nivolumab versus Everolimus in Advanced Renal-Cell Carcinoma. The New England journal of medicine. 2015;373(19):1803–13. 10.1056/NEJMoa1510665 . 26406148 PMC5719487

[13] Motzer RJ, Hutson TE, Glen H, Michaelson MD, Molina A, Eisen T, et al Lenvatinib, everolimus, and the combination in patients with metastatic renal cell carcinoma: a randomised, phase 2, open-label, multicentre trial. The lancet oncology. 2015;16(15):1473–82. 10.1016/S1470-2045(15)00290-9 . 26482279

[14] Comprehensive molecular characterization of clear cell renal cell carcinoma. Nature. 2013;499(7456):43–9. Epub 2013/06/25. 10.1038/nature12222 ; PubMed Central PMCID: PMCPmc3771322. 23792563 PMC3771322

[15] Brugarolas J. Molecular genetics of clear-cell renal cell carcinoma. Journal of clinical oncology: official journal of the American Society of Clinical Oncology. 2014;32(18):1968–76. 10.1200/JCO.2012.45.2003 24821879 PMC4050206

[16] Behbahani TE, Thierse C, Baumann C, Holl D, Bastian PJ, von Ruecker A, et al Tyrosine kinase expression profile in clear cell renal cell carcinoma. World J Urol. 2012;30(4):559–65. 10.1007/s00345-011-0767-z . 21969129

[17] Pena-Llopis S, Vega-Rubin-de-Celis S, Liao A, Leng N, Pavia-Jimenez A, Wang S, et al BAP1 loss defines a new class of renal cell carcinoma. Nature genetics. 2012;44(7):751–9. 10.1038/ng.2323 22683710 PMC3788680

[18] Carlo MI, Molina AM, Lakhman Y, Patil S, Woo K, DeLuca J, et al A Phase Ib Study of BEZ235, a Dual Inhibitor of Phosphatidylinositol 3-Kinase (PI3K) and Mammalian Target of Rapamycin (mTOR), in Patients With Advanced Renal Cell Carcinoma. The oncologist. 2016 10.1634/theoncologist.2016-0145 . 27286790 PMC4943396

[19] Powles T, Lackner MR, Oudard S, Escudier B, Ralph C, Brown JE, et al Randomized Open-Label Phase II Trial of Apitolisib (GDC-0980), a Novel Inhibitor of the PI3K/Mammalian Target of Rapamycin Pathway, Versus Everolimus in Patients With Metastatic Renal Cell Carcinoma. Journal of clinical oncology: official journal of the American Society of Clinical Oncology. 2016;34(14):1660–8. 10.1200/JCO.2015.64.8808 . 26951309 PMC5569691

[20] Gerlinger M, Rowan AJ, Horswell S, Larkin J, Endesfelder D, Gronroos E, et al Intratumor heterogeneity and branched evolution revealed by multiregion sequencing. The New England journal of medicine. 366(10):883–92. Epub 2012/03/09. 10.1056/NEJMoa1113205 . 22397650 PMC4878653

[21] Gerlinger M, Horswell S, Larkin J, Rowan AJ, Salm MP, Varela I, et al Genomic architecture and evolution of clear cell renal cell carcinomas defined by multiregion sequencing. Nature genetics. 2014;46(3):225–33. 10.1038/ng.2891 . 24487277 PMC4636053

[22] Wu P, Nielsen TE, Clausen MH. FDA-approved small-molecule kinase inhibitors. Trends Pharmacol Sci. 2015;36(7):422–39. 10.1016/j.tips.2015.04.005 . 25975227

[23] Geiss GK, Bumgarner RE, Birditt B, Dahl T, Dowidar N, Dunaway DL, et al Direct multiplexed measurement of gene expression with color-coded probe pairs. Nature biotechnology. 2008;26(3):317–25. 10.1038/nbt1385 . 18278033

[24] Beard RE, Abate-Daga D, Rosati SF, Zheng Z, Wunderlich JR, Rosenberg SA, et al Gene expression profiling using nanostring digital RNA counting to identify potential target antigens for melanoma immunotherapy. Clinical cancer research: an official journal of the American Association for Cancer Research. 2013;19(18):4941–50. 10.1158/1078-0432.CCR-13-1253 24021875 PMC3778100

[25] Sestak I, Dowsett M, Zabaglo L, Lopez-Knowles E, Ferree S, Cowens JW, et al Factors predicting late recurrence for estrogen receptor-positive breast cancer. Journal of the National Cancer Institute. 2013;105(19):1504–11. 10.1093/jnci/djt244 24029245 PMC3787911

[26] Dowsett M, Sestak I, Lopez-Knowles E, Sidhu K, Dunbier AK, Cowens JW, et al Comparison of PAM50 risk of recurrence score with oncotype DX and IHC4 for predicting risk of distant recurrence after endocrine therapy. J Clin Oncol. 2013;31(22):2783–90. 10.1200/JCO.2012.46.1558 . 23816962

[27] Lohavanichbutr P, Mendez E, Holsinger FC, Rue TC, Zhang Y, Houck J, et al A 13-gene signature prognostic of HPV-negative OSCC: discovery and external validation. Clinical cancer research: an official journal of the American Association for Cancer Research. 2013;19(5):1197–203. 10.1158/1078-0432.CCR-12-2647 23319825 PMC3593802

[28] Kulkarni MM. Digital multiplexed gene expression analysis using the NanoString nCounter system. Curr Protoc Mol Biol. 2011;Chapter 25:Unit25B.10. 10.1002/0471142727.mb25b10s94 . 21472696

[29] Love MI, Huber W, Anders S. Moderated estimation of fold change and dispersion for RNA-seq data with DESeq2. Genome Biol. 2014;15(12):550 10.1186/s13059-014-0550-8 25516281 PMC4302049

[30] Hochberg Y, Benjamini Y. More powerful procedures for multiple significance testing. Statistics in medicine. 1990;9(7):811–8. . 2218183 10.1002/sim.4780090710

[31] Mosch B, Reissenweber B, Neuber C, Pietzsch J. Eph receptors and ephrin ligands: important players in angiogenesis and tumor angiogenesis. J Oncol. 2010;2010:135285 10.1155/2010/135285 20224755 PMC2836134

[32] Khansaard W, Techasen A, Namwat N, Yongvanit P, Khuntikeo N, Puapairoj A, et al Increased EphB2 expression predicts cholangiocarcinoma metastasis. Tumour Biol. 2014;35(10):10031–41. 10.1007/s13277-014-2295-0 . 25012246

[33] Gao Q, Liu W, Cai J, Li M, Gao Y, Lin W, et al EphB2 promotes cervical cancer progression by inducing epithelial-mesenchymal transition. Hum Pathol. 2014;45(2):372–81. 10.1016/j.humpath.2013.10.001 . 24439224

[34] Jubb AM, Zhong F, Bheddah S, Grabsch HI, Frantz GD, Mueller W, et al EphB2 is a prognostic factor in colorectal cancer. Clinical cancer research: an official journal of the American Association for Cancer Research. 2005;11(14):5181–7. 10.1158/1078-0432.CCR-05-0143 . 16033834

[35] Kandouz M, Haidara K, Zhao J, Brisson ML, Batist G. The EphB2 tumor suppressor induces autophagic cell death via concomitant activation of the ERK1/2 and PI3K pathways. Cell Cycle. 2010;9(2):398–407. . 20046096 10.4161/cc.9.2.10505

[36] Lugli A, Spichtin H, Maurer R, Mirlacher M, Kiefer J, Huusko P, et al EphB2 expression across 138 human tumor types in a tissue microarray: high levels of expression in gastrointestinal cancers. Clinical cancer research: an official journal of the American Association for Cancer Research. 2005;11(18):6450–8. 10.1158/1078-0432.CCR-04-2458 . 16166419

[37] Mao W, Luis E, Ross S, Silva J, Tan C, Crowley C, et al EphB2 as a therapeutic antibody drug target for the treatment of colorectal cancer. Cancer research. 2004;64(3):781–8. . 14871799 10.1158/0008-5472.can-03-1047

[38] Pal SK, He M, Tong T, Wu H, Liu X, Lau C, et al RNA-seq Reveals Aurora Kinase Driven-mTOR Pathway Activation in Patients with Sarcomatoid Metastatic Renal Cell Carcinoma. Mol Cancer Res. 2014 10.1158/1541-7786.MCR-14-0352 . 25183163 PMC4608366

[39] Dai J, Sultan S, Taylor SS, Higgins JM. The kinase haspin is required for mitotic histone H3 Thr 3 phosphorylation and normal metaphase chromosome alignment. Genes Dev. 2005;19(4):472–88. 10.1101/gad.1267105 15681610 PMC548948

[40] Huertas D, Soler M, Moreto J, Villanueva A, Martinez A, Vidal A, et al Antitumor activity of a small-molecule inhibitor of the histone kinase Haspin. Oncogene. 2012;31(11):1408–18. 10.1038/onc.2011.335 21804608 PMC3312407

[41] Hildebrandt MA, Tan W, Tamboli P, Huang M, Ye Y, Lin J, et al Kinome expression profiling identifies IKBKE as a predictor of overall survival in clear cell renal cell carcinoma patients. Carcinogenesis. 2012;33(4):799–803. 10.1093/carcin/bgs018 22266464 PMC3324439

[42] Peri S, Devarajan K, Yang DH, Knudson AG, Balachandran S. Meta-analysis identifies NF-kappaB as a therapeutic target in renal cancer. PLoS One. 2013;8(10):e76746 10.1371/journal.pone.0076746 24116146 PMC3792024

[43] Chung S, Suzuki H, Miyamoto T, Takamatsu N, Tatsuguchi A, Ueda K, et al Development of an orally-administrative MELK-targeting inhibitor that suppresses the growth of various types of human cancer. Oncotarget. 2012;3(12):1629–40. 23283305 10.18632/oncotarget.790 PMC3681500

[44] Joshi K, Banasavadi-Siddegowda Y, Mo X, Kim SH, Mao P, Kig C, et al MELK-dependent FOXM1 phosphorylation is essential for proliferation of glioma stem cells. Stem Cells. 2013;31(6):1051–63. 10.1002/stem.1358 23404835 PMC3744761

[45] Gu C, Banasavadi-Siddegowda YK, Joshi K, Nakamura Y, Kurt H, Gupta S, et al Tumor-specific activation of the C-JUN/MELK pathway regulates glioma stem cell growth in a p53-dependent manner. Stem Cells. 2013;31(5):870–81. 10.1002/stem.1322 23339114 PMC4132653

[46] Minata M, Gu C, Joshi K, Nakano-Okuno M, Hong C, Nguyen CH, et al Multi-kinase inhibitor C1 triggers mitotic catastrophe of glioma stem cells mainly through MELK kinase inhibition. PLoS One. 2014;9(4):e92546 10.1371/journal.pone.0092546 24739874 PMC3989164

[47] Feng X, Lu X, Man X, Zhou W, Jiang LQ, Knyazev P, et al Overexpression of Csk-binding protein contributes to renal cell carcinogenesis. Oncogene. 2009;28(37):3320–31. 10.1038/onc.2009.185 . 19581936

[48] Cybulski C, Gorski B, Huzarski T, Masojc B, Mierzejewski M, Debniak T, et al CHEK2 is a multiorgan cancer susceptibility gene. Am J Hum Genet. 2004;75(6):1131–5. 10.1086/426403 15492928 PMC1182149

[49] Stricker TP, Morales La Madrid A, Chlenski A, Guerrero L, Salwen HR, Gosiengfiao Y, et al Validation of a prognostic multi-gene signature in high-risk neuroblastoma using the high throughput digital NanoString nCounter™ system. Mol Oncol. 2014;8(3):669–78. 10.1016/j.molonc.2014.01.010 24560446 PMC4004665

[50] Rini B, Goddard A, Knezevic D, Maddala T, Zhou M, Aydin H, et al A 16-gene assay to predict recurrence after surgery in localised renal cell carcinoma: development and validation studies. The lancet oncology. 2015;16(6):676–85. 10.1016/S1470-2045(15)70167-1 . 25979595

[51] Schutz FA, Pomerantz MM, Gray KP, Atkins MB, Rosenberg JE, Hirsch MS, et al Single nucleotide polymorphisms and risk of recurrence of renal-cell carcinoma: a cohort study. The lancet oncology. 2013;14(1):81–7. 10.1016/S1470-2045(12)70517-X 23219378 PMC3769687

[52] Sültmann H, von Heydebreck A, Huber W, Kuner R, Buness A, Vogt M, et al Gene expression in kidney cancer is associated with cytogenetic abnormalities, metastasis formation, and patient survival. Clin Cancer Res. 2005;11(2 Pt 1):646–55. . 15701852

