# Supplementary material for: Kinase Gene Expression Profiling of Metastatic Clear Cell Renal Cell Carcinoma Tissue Identifies Potential New Therapeutic Targets
Source: PLoS One. 2016 Aug 30;11(8):e0160924. doi: 10.1371/journal.pone.0160924 (PMC5004806; doi:10.1371/journal.pone.0160924)
Supplement: S3 Table — (DOCX) [file pone.0160924.s003.docx]

**S 3 Table**: Comparison of expression of all kinase genes between primary tumors from metastatic and non-metastatic patients^*^

| **Genes** | **baseMean** | **p-value** | **adjusted p-value** |
| --- | --- | --- | --- |
| PLK1.5347 | 292.0170653 | 3.06E-20 | 1.59E-17 |
| BUB1.699 | 306.2341715 | 4.87E-18 | 1.61E-15 |
| BUB1B.701 | 208.9582476 | 4.59E-17 | 1.26E-14 |
| NEK2.4751 | 134.4702346 | 8.07E-16 | 1.52E-13 |
| PKMYT1.9088 | 126.8593633 | 3.37E-14 | 4.62E-12 |
| AURKA.6790 | 353.7879497 | 3.39E-10 | 1.85E-08 |
| IKBKE.9641 | 496.7497848 | 3.98E-10 | 2.12E-08 |
| EPHB2.2048 | 389.0931124 | 1.69E-09 | 7.51E-08 |
| PRKCE.5581 | 1034.047945 | 3.53E-09 | 1.42E-07 |
| TYRO3.7301 | 421.2091912 | 1.31E-06 | 1.96E-05 |
| PLK4.10733 | 167.5924211 | 2.94E-06 | 3.82E-05 |
| ROS1.6098 | 3.64083759 | 3.60E-06 | 4.50E-05 |
| CDC7.8317 | 251.725834 | 2.29E-05 | 0.000202497 |
| CHEK1.1111 | 385.3813326 | 3.39E-05 | 0.000281069 |
| KALRN.8997 | 1437.020147 | 3.99E-05 | 0.000322178 |
| DYRK4.8798 | 799.8344802 | 0.000391114 | 0.002043135 |
| PDGFRA.5156 | 940.8496297 | 0.001240444 | 0.005219818 |
| BLK.640 | 31.74960755 | 0.003490822 | 0.012053441 |
| IRAK3.11213 | 974.4304795 | 0.006061049 | 0.018805998 |
| ROR2.4920 | 368.5028618 | 0.010482842 | 0.029193302 |
| TEC.7006 | 173.9342453 | 0.01432477 | 0.037496982 |
| BTK.695 | 618.8214534 | 0.032809427 | 0.073328612 |
| AATK.9625 | 250.5629034 | 0.099008711 | 0.176238809 |
| ADCK1.57143 | 281.2929891 | 0.410030272 | 0.525165832 |
| EPHA3.2042 | 1021.112815 | 0.426112208 | 0.540750246 |
| PRKCA.5578 | 2168.054189 | 0.482597334 | 0.593622703 |
| FES.2242 | 949.7965792 | 0.566257186 | 0.669159184 |
| MAPK11.5600 | 891.4717632 | 0.656886427 | 0.744419596 |
| PRKCQ.5588 | 420.7596441 | 0.702573088 | 0.780578049 |
| EPHB3.2049 | 280.0622889 | 0.722668076 | 0.796126344 |
| PASK.23178 | 458.7715619 | 0.897421294 | 0.926475385 |
| CAMK4.814 | 64.74672799 | 0.907125477 | 0.933108575 |
| MAPK12.6300 | 899.4799728 | 0.920539963 | 0.942428369 |

^*^Comparing TCGA patients who had metastasis at baseline (n=79) vs. those who did not develop metastasis for at least 2 years (n=187); baseMean (DESeq2 generated normalized mean count).
