# Supplementary material for: Kinase Gene Expression Profiling of Metastatic Clear Cell Renal Cell Carcinoma Tissue Identifies Potential New Therapeutic Targets
Source: PLoS One. 2016 Aug 30;11(8):e0160924. doi: 10.1371/journal.pone.0160924 (PMC5004806; doi:10.1371/journal.pone.0160924)
Supplement: S4 Table — (DOCX) [file pone.0160924.s004.docx]

**S 4 Table**: Comparison of expression of all kinase genes between primary tumors from patients without metastasis at resection and did not recur vs. those who recurred^*^

| **Genes** | **baseMean** | **p-value** | **adjusted p-value** |
| --- | --- | --- | --- |
| PLK1.5347 | 280.7094196 | 7.15E-24 | 4.73E-21 |
| NEK2.4751 | 130.7511871 | 3.63E-19 | 1.02E-16 |
| BUB1B.701 | 197.9035811 | 6.08E-16 | 9.61E-14 |
| PKMYT1.9088 | 119.7195365 | 4.73E-13 | 4.59E-11 |
| AURKA.6790 | 345.7203584 | 3.69E-12 | 3.15E-10 |
| BUB1.699 | 266.9655725 | 2.55E-11 | 1.89E-09 |
| EPHB2.2048 | 363.3853215 | 4.12E-09 | 2.05E-07 |
| CDC7.8317 | 250.273927 | 3.15E-07 | 9.94E-06 |
| PDGFRA.5156 | 998.3886383 | 3.79E-07 | 1.16E-05 |
| CHEK1.1111 | 383.3490676 | 2.80E-06 | 6.52E-05 |
| ROR2.4920 | 404.1174895 | 4.04E-06 | 8.84E-05 |
| TYRO3.7301 | 404.2397428 | 9.25E-06 | 0.000179936 |
| PLK4.10733 | 162.9510583 | 2.78E-05 | 0.000450029 |
| ROS1.6098 | 2.885465916 | 0.000117077 | 0.001447516 |
| PRKCA.5578 | 2306.826529 | 0.000151269 | 0.001781627 |
| IKBKE.9641 | 441.7227848 | 0.000524544 | 0.004842197 |
| PRKCE.5581 | 1111.840171 | 0.005446771 | 0.02877124 |
| DYRK4.8798 | 781.2239329 | 0.0104575 | 0.046024781 |
| BLK.640 | 28.60826568 | 0.089673364 | 0.214624364 |
| PRKCQ.5588 | 409.3258062 | 0.230945012 | 0.407421461 |
| MAPK11.5600 | 903.1396822 | 0.285168248 | 0.468861829 |
| BTK.695 | 582.6964313 | 0.305825644 | 0.491764357 |
| EPHA3.2042 | 1074.059868 | 0.34837381 | 0.533747422 |
| ADCK1.57143 | 284.1287287 | 0.359069765 | 0.545238694 |
| CAMK4.814 | 66.22443469 | 0.422103716 | 0.603704191 |
| KALRN.8997 | 1554.5623 | 0.423424883 | 0.604879188 |
| PASK.23178 | 458.9465951 | 0.519687805 | 0.684867917 |
| AATK.9625 | 264.0157952 | 0.533271832 | 0.695891834 |
| EPHB3.2049 | 281.4412743 | 0.618701579 | 0.75908556 |
| FES.2242 | 949.7337354 | 0.714027519 | 0.824866516 |
| IRAK3.11213 | 1035.479504 | 0.792537183 | 0.874519076 |
| TEC.7006 | 180.9982922 | 0.809174315 | 0.885897983 |
| MAPK12.6300 | 912.3044523 | 0.851604917 | 0.911423134 |

^*^Comparing TCGA patients who did not have metastases at the time of resection and were recurrence free for 2+ years (n=187) vs. those who did not have metastases at the time of resection but later recurred (n=28); baseMean (DESeq2 generated normalized mean count).
