# Supplementary material for: Kinase Gene Expression Profiling of Metastatic Clear Cell Renal Cell Carcinoma Tissue Identifies Potential New Therapeutic Targets
Source: PLoS One. 2016 Aug 30;11(8):e0160924. doi: 10.1371/journal.pone.0160924 (PMC5004806; doi:10.1371/journal.pone.0160924)
Supplement: S5 Table — (DOCX) [file pone.0160924.s005.docx]

**S 5 Table**: Comparison of kinase gene expression between primary tumors from metastatic and non-metastatic patients at baseline^*^

| **Genes** | **baseMean** | **p-value** | **adjusted p-value** |
| --- | --- | --- | --- |
| PLK1.5347 | 296.0457865 | 2.38E-11 | 6.11E-09 |
| BUB1.699 | 305.0702043 | 5.53E-11 | 1.12E-08 |
| NEK2.4751 | 134.7448277 | 2.06E-09 | 2.25E-07 |
| PKMYT1.9088 | 127.9792568 | 9.95E-09 | 8.50E-07 |
| BUB1B.701 | 217.1315566 | 7.21E-08 | 4.34E-06 |
| IKBKE.9641 | 490.9034348 | 7.71E-08 | 4.57E-06 |
| PRKCE.5581 | 1019.701344 | 1.31E-07 | 7.15E-06 |
| AURKA.6790 | 350.417601 | 2.43E-07 | 1.15E-05 |
| EPHB2.2048 | 395.065786 | 4.05E-06 | 0.000101778 |
| PLK4.10733 | 163.6415794 | 2.23E-05 | 0.000367837 |
| KALRN.8997 | 1406.181347 | 0.000100242 | 0.001134683 |
| TYRO3.7301 | 423.7380396 | 0.000188902 | 0.001800797 |
| BLK.640 | 28.63650797 | 0.000516022 | 0.003877326 |
| DYRK4.8798 | 793.3120927 | 0.001850934 | 0.01009925 |
| CHEK1.1111 | 385.6268453 | 0.002144145 | 0.011272588 |
| CDC7.8317 | 255.5214468 | 0.002564082 | 0.012919856 |
| ROS1.6098 | 3.953632703 | 0.004790007 | 0.020720416 |
| IRAK3.11213 | 960.0589226 | 0.010440255 | 0.03724401 |
| BTK.695 | 586.8544689 | 0.011418499 | 0.039666649 |
| TEC.7006 | 172.6757292 | 0.0191853 | 0.05865717 |
| AATK.9625 | 258.416727 | 0.03208428 | 0.085410015 |
| PDGFRA.5156 | 1000.239238 | 0.035574751 | 0.092424174 |
| ADCK1.57143 | 286.7812195 | 0.179187366 | 0.305174918 |
| ROR2.4920 | 413.6634376 | 0.206203462 | 0.338184935 |
| EPHA3.2042 | 1026.715834 | 0.347341424 | 0.490522406 |
| CAMK4.814 | 59.9951048 | 0.423912964 | 0.563692841 |
| PASK.23178 | 455.7815975 | 0.773377684 | 0.842330696 |
| MAPK11.5600 | 887.6018599 | 0.823426903 | 0.877952756 |
| PRKCQ.5588 | 418.5145693 | 0.82582959 | 0.879725836 |
| MAPK12.6300 | 892.6982231 | 0.866374569 | 0.908388675 |
| EPHB3.2049 | 281.5460013 | 0.943624285 | 0.960356098 |
| PRKCA.5578 | 2214.193044 | 0.97957126 | 0.98494986 |
| FES.2242 | 957.7169783 | 0.993352747 | 0.994436136 |

^*^Comparing TCGA patients who had metastasis at baseline (n=79) vs. those who did not (n=418). baseMean (DESeq2 generated normalized mean count).
